# Renal oncocytoma in a kidney transplant patient: the imaging features on contrast-enhanced ultrasonography (CEUS): a case report

**DOI:** 10.1590/1678-4685-JBN-3787

**Published:** 2018-04-19

**Authors:** Lucia Alejandra Alfaro Villanueva, Maira Knust, Leonardo Quintella, José Hermógenes R Suassuna, Nordeval C Araújo

**Affiliations:** 1 Hospital Universitário Pedro Ernesto, Vila Isabel, Rio de Janeiro, RJ, Brasil.

**Keywords:** Adenoma, Oxyphilic, Ultrasonography, Kidney Neoplasms, Microbubbles, Adenoma oxifílico, ultrassonografia, neoplasias renais, microbolhas

## Abstract

Renal oncocytoma is an infrequently reported renal neoplasm, often asymptomatic, which usually behaves as a benign entity and is identified accidentally on radiological imaging. Transplant patients under long-term immunosuppressive drugs have a high prevalence of cancers, such as skin cancers, lymphoproliferative disorders, and renal carcinomas. We present a case report of an asymptomatic renal oncocytoma in a kidney transplant recipient presenting persistent hematuria. The features of computed tomography and contrast-enhanced ultrasound (CEUS) are presented. This was the first time we used CEUS in a transplant kidney recipient presenting a renal mass, allowing the real-time visualization of contrast-enhancement patterns during all vascular phases for the differential diagnosis of renal tumors. Although the pattern of intense vascularization could mislead to an early judgment as a malignant lesion, it could help to exclude other renal lesions without inducing nephrotoxicity.

## Resumo

O oncocitoma renal é uma neoplasia renal raramente relatada, muitas vezes assintomática, que geralmente se comporta como uma entidade benigna e é identificada acidentalmente em imagens radiológicas. Pacientes transplantados em regimes imunossupressores de longa duração apresentam alta prevalência de neoplasias tais como câncer de pele, distúrbios linfoproliferativos e carcinomas renais. Apresentamos o relato de um caso de oncocitoma renal assintomático em receptor de transplante renal com hematúria persistente. São apresentados os achados de imagens de tomografia computadorizada e ultrassonografia (US) com contraste. Foi a primeira vez que utilizamos a US com contraste em um receptor de transplante renal que apresentava massa renal, permitindo a visualização em tempo real dos padrões de realce do contraste em todas as fases vasculares para o diagnóstico diferencial dos tumores renais. Embora o padrão de vascularização intensa possa induzir uma avaliação precoce de lesão maligna, o exame ajuda a excluir outras lesões renais sem induzir nefrotoxicidade.

## Introduction

Renal oncocytoma is a tumor of renal tubular origin, accounting for 3-7% of all solid renal tumors.^1^ It is an infrequently reported renal neoplasm, which usually behaves as a benign entity and consists of a pure population of oncocytes. Males are affected more commonly and the mean age of presentation is in the 6-7th decade.^2^ Preoperative diagnosis is often difficult as the lesion can mimic renal cell carcinoma both clinically and radiologically. Transplant patients under long-term immunosuppressive drugs have an unusually high prevalence of cancers such as skin cancers, lymphoproliferative disorders, and renal carcinomas.^3^ In this paper, we present a case of renal oncocytoma in a kidney transplant patient presenting with asymptomatic microscopic hematuria. Diagnostic imaging included conventional sonography, computed tomography, and contrast-enhanced ultrasonography (CEUS). The aim of this case report was to highlight the features of oncocytoma enhancement on CEUS. Additionally, this case illustrates the difficulty in making accurate preoperative diagnoses despite the use of modern scanning facilities.

## Case presentation

**Figure 1:**
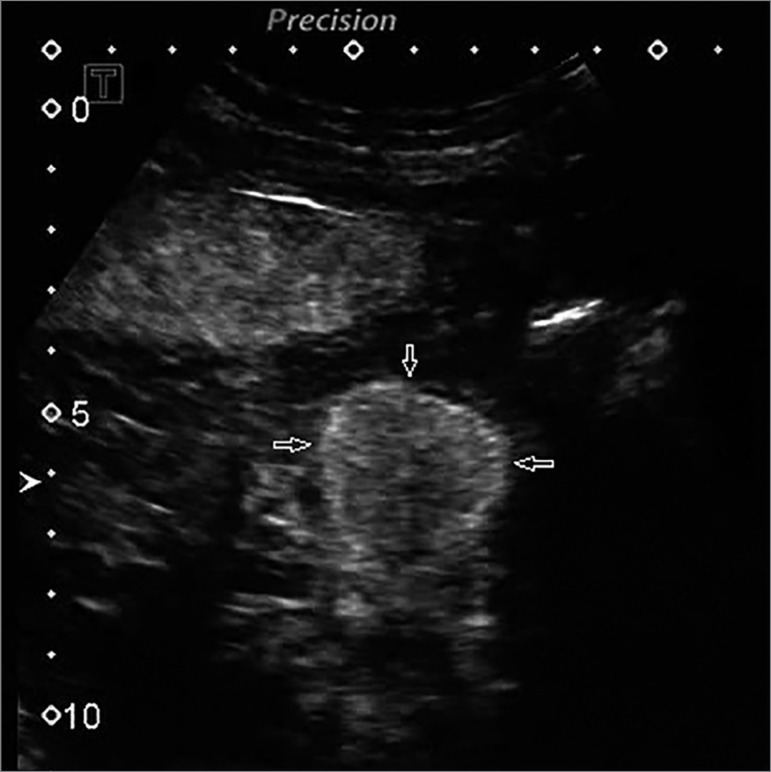
CEUS image showing hypervascular mass in relation to the remained parenchyma of the native kidney with heterogeneous enhancement and pseudocapsule sign (arrows).

**Figure 2:**
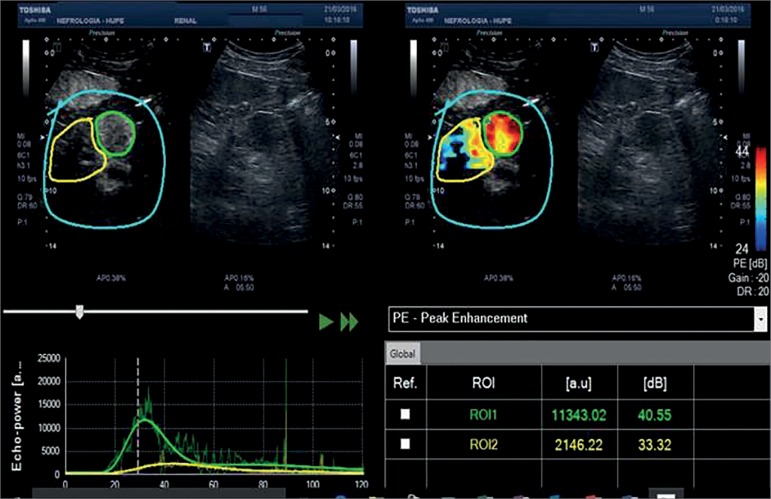
(CEUS) - Quantitative analysis with time-intensity curve. In the arterial phase, the mass was considered hypervascular when compared to the remaining parenchyma.

In 2012, a 56-year old male received a cadaveric renal transplant, with 2 human leukocyte antigen (HLA) mismatches, for chronic renal failure related to hypertension. He was an ex-smoker who had been undergoing hemodialysis for four years. There were no records of blood transfusions or previous kidney transplantation. The cold ischemia time was 17 hours. The immunosuppressive regimen for induction consisted of Basiliximab. Maintenance immunosuppression included tacrolimus 0.1 mg/kg/day, mycophenolate, and prednisone. Immediately after transplantation, he developed delayed graft function with the need for hemodialysis for one week, later achieving a stable renal function; he currently has a serum creatinine of 1.4 mg/dl, which translates to an estimated glomerular filtration rate of 55 mL/min/1.73m^2^. Four years after kidney transplant, at an outpatient medical follow-up, he presented asymptomatic microscopic non-glomerular hematuria confirmed by the absence of erythrocytic dysmorphism in the phase-contrast microscopy of the urine. The physical examination was normal. The serum creatinine was 1.7 mg/dL. A sonogram showed a solid mass on the left native kidney. Using a 3.5 MHz convex transducer (Aplio 400; Toshiba; Tokyo, Japan), a CEUS with Sonovue^®^ (Bracco Int; Milan, Italy) bolus of 2.4 mL injected using a 20-gauge intravenous cannula, followed by a 10 mL saline flush was performed. The examination was performed using contrast harmonic imaging at a low mechanical index of 0,1. The exam was documented by digitally storing the images over 60 s in DICOM format. The images showed a hypervascular mass in relation to the remaining parenchyma of the native kidney with heterogeneous enhancement and pseudocapsule sign (Figure 1). Quantitative analysis with time-intensity curve was used to calculate the amount of enhancement in the mass and remaining parenchyma of the native kidney. Accordingly, in the arterial phase, the mass was considered hypervascular when compared to the remaining parenchyma (Figure 2).

**Figure 3:**
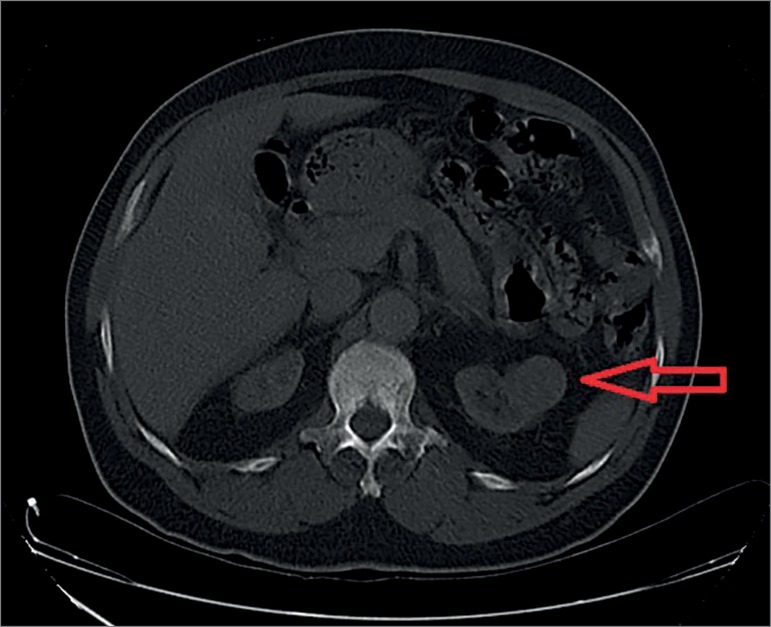
Renal oncocytoma - CT scan exhibiting the typical imaging morphology of a solid, homogenous, unilateral lesion with isodensity as compared to the normal adjacent renal parenchyma.

A CT scan confirmed the presence of a unilateral isodense renal nodule of the upper pole (Figure 3).

**Figure 4:**
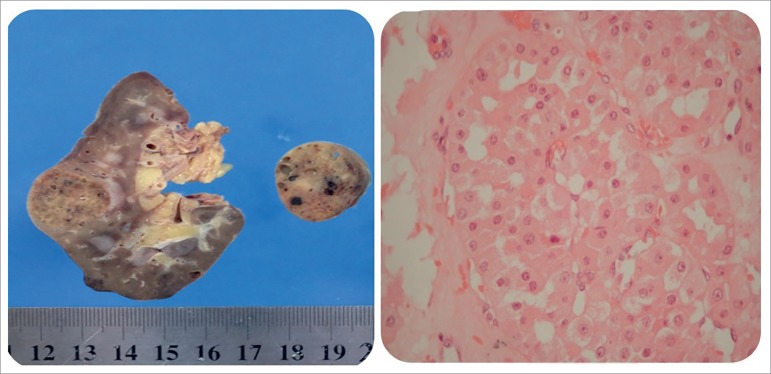
Left - The 2.5 cm diameter tumor was reddish-brown, lighter than cortex, with well-circumscribed borders that mimics a capsule, and without central scar. In addition, there were multiple coalescing small grayish nodules scattered throughout the mass. Right - microscopic findings showed numerous tubular cells with abundant granular cytoplasm and large nuclei.

The patient underwent total removal of the left kidney. The 2.5 cm diameter tumor was reddish-brown with well-circumscribed borders that mimic a capsule. It also revealed multiple nodules of 2.5 cm, 0.7 cm and 0.5 cm in diameter and supported the diagnosis of multicentric oncocytoma. The microscopic findings showed numerous tubular cells with abundant granular cytoplasm and large nuclei (Figure 4).

Following nephrectomy, the patient was followed-up for six months and showed normal renal function and no more microscopic hematuria.

Since we began to perform CEUS in our facility as clinical research, the ethics committee approved the study according to local legal requirements and informed consent for contrast use and the report of the case was obtained from the patient.

## Discussion

Hematuria has a prevalence of 12% in the post-renal transplant patient population.^4^

In one series, the most prevalent etiology for hematuria was urologic malignancy.^5^

Renal cell cancers represent 5-6% of all cancers.^3^ While the overall incidence of malignancy after renal transplantation is 3-5 times higher than in the general population, the risk of renal cell carcinoma development is 15-100 times higher.^6^ Longer exposure to immunosuppressive medications has been implicated as a possible risk factor for cancer after transplantation.^6^

Oncocytoma is the most common benign renal parenchymal solid tumor.^7^ This tumor represents a distinct entity, reported for the first time by Zippel in 1942.^8^ It was not until 1976 that renal oncocytoma was reconsidered by Klein and Valensi.^9^ These authors published a retrospective study containing a case series of patients with oncocytomas, and highlighted the benign course of the disease. It accounts for 3-7% of renal tumors^2^ and occurs between 40 and 80 years of age, with a male predominance and a high prevalence of smokers.^9^ Usually, these lesions are accidental findings in exams for other reasons, but hematuria is the most common complaint in symptomatic patients. Since these tumors are often asymptomatic, clinicians should perform a renal sonogram once a year to address this issue.^10^

On gross appearance, these tumors are cortically localized, light brown or tan, homogeneous, well circumscribed and with a commonly seen central scar.^1^ The current case, while quite similar to the most common presentation, had no central scar. The central scar represents an avascular area that develops as the tumor grows. Oncocytoma is comprised solely of oncocytes.^10^ The origin of these cells is considered to be the intercallary cell of the cortical portion of the collecting tubule.^1^

Radiographically, renal oncocytomas appear as solid masses which are vascular on angiography; the findings include a spoke-wheel pattern and a homogeneous nephrogram.^10^ Nevertheless, renal cell carcinomas may also have these features. On CT scans, oncocytomas are typically hypervascular, homogeneous, and present with a characteristic central scar.

The contrast-enhancement ultrasonography (CEUS) is a well-established technique for imaging of the liver and other organs, which uses ultrasound contrast agents to improve the visualization and characterization of anatomic structures and lesions. The use of CEUS in renal tumors can identify different enhancement patterns and areas of perfusion. CEUS has been advocated as the imaging modality of choice to evaluate patients with renal impairment, given the absence of nephrotoxicity.^11^ The method has been claimed to contribute to the differential diagnosis of renal tumor histotypes.^12^ However, since the hypervascular pattern observed in the present case has also been reported in renal cell carcinoma (RCC), the differentiation of oncocytomas from RCC, by CEUS, is often difficult because of the overlap of imaging features.^13^ Moreover, the pseudocapsule surrounding the mass seen on CEUS is not a pathognomonic feature of oncocytoma, since it has been reported in renal cell carcinoma as well.^13^ Therefore, CEUS, like computed tomography, is not able to differentiate oncocytoma from RCC.^14^ This case highlights the difficulties in making a preoperative diagnosis despite the use of modern scanning.

To the best of our knowledge, this is the first case of a renal oncocytoma in an asymptomatic renal transplant patient with microscopic hematuria in which a hypervascular mass with heterogeneous enhancement and pseudocapsule was shown by using CEUS, suggesting malignancy. Furthermore, only four cases of renal transplant recipients with oncocytomas in native kidneys have been reported in the literature.^15^

To conclude, a case of asymptomatic hematuria due to renal oncocytoma in a native kidney was observed in a renal transplant patient. Patient follow-up was unremarkable six months after surgery. The use of CEUS should be considered an important tool to evaluate the morphology and vascularization pattern of focal lesions with no risk of nephrotoxicity; however, it is not yet possible to differentiate benign from malignant tumors.
